# Estimation of the Genetic Parameters for Semen Traits in Spanish Dairy Sheep

**DOI:** 10.3390/ani9121147

**Published:** 2019-12-13

**Authors:** Rocío Pelayo, Manuel Ramón, Itsasne Granado-Tajada, Eva Ugarte, Malena Serrano, Beatriz Gutiérrez-Gil, Juan-José Arranz

**Affiliations:** 1 Departamento de Producción Animal, Facultad de Veterinaria, Universidad de León, Campus de Vegazana s/n, 24071 León, Spain; rpelg@unileon.es (R.P.); bgutg@unileon.es (B.G.-G.); 2 Instituto Regional de Investigación y Desarrollo Agroalimentario y Forestal de Castilla La Mancha (IRIAF), CERSYRA de Valdepeñas, 13300 Ciudad Real, Spain; m.ramon.fernandez@gmail.com; 3 NEIKER, Instituto Vasco de Investigación y Desarrollo Agrario, Campus Agroalimentario de Arkaute, Apdo 46, 01080 Vitoria-Gasteiz, Spain; igranado@neiker.eus (I.G.-T.); eugarte@neiker.eus (E.U.); 4 INIA. Departamento de Mejora genética Animal. Ctra. de la Coruña km 7.5, 28040 Madrid, Spain; malena@inia.es

**Keywords:** semen traits, sheep, genetic parameters, heritability

## Abstract

**Simple Summary:**

The limited studies addressing the estimation of genetic parameters for ram semen traits in different breeds show a wide variation, highlighting the importance of studying these traits for individual breeds. Therefore, this work aimed to estimate genetic parameters for traits related to semen production and quality in five dairy sheep breeds. For that, ejaculates of rams from Assaf, Churra, Latxa Cara Negra, Latxa Cara Rubia, and Manchega breeds were analyzed. Estimates of the genetic covariance structure were obtained with multiple-trait animal models using the average information REML (restricted maximum likelihood) method in the BLUPF90 family of programs. Repeatability estimates for all the traits were also calculated. Heritability estimates were of low to moderate magnitude, although the estimates differed among the breeds. The estimated genetic correlations among the three semen traits showed adequate precision only in the Manchega (MAN) breed. The heritability estimates reported here suggest that improvement of these traits may be achieved by genetic selection.

**Abstract:**

This work aimed to estimate genetic parameters for traits related to semen production and quality in Spanish dairy sheep breeds. For that, ejaculates of rams from Assaf, Churra, Latxa Cara Negra, Latxa Cara Rubia, and Manchega breeds were analyzed to measure volume, semen concentration, and motility. Estimates of variance components were obtained with multiple-trait animal models using the average information REML method in the BLUPF90 family of programs. Repeatability estimates for all the traits were also calculated, with values ranging from 0.077 to 0.304 for the motility and the semen concentration traits, respectively. Heritability estimates were of low to moderate magnitude, ranging from 0.014 (motility in Latxa Cara Rubia) to 0.198 (volume in Churra), although the estimates differed among the breeds. The estimated genetic correlations among the three semen traits showed adequate precision only in the MAN breed. The heritability estimates for the semen traits reported in the present paper suggest an adequate response to selection. The practical extension of these results to the other breeds studied here will be secondary to the estimation of more reliable genetic correlations in these breeds.

## 1. Introduction

Of the countries in the European Union, Spain has the second-highest number of dairy sheep, with a population of up to 16 million in 2018 [1]. Dairy sheep farms are an essential factor in the maintenance of rural development through environmentally friendly and sustainable production systems, which provide a broad diversity of high-quality products, including artisanal mature cheese and suckling lamb meat [2]. For many years, sheep milk production in Spain has been focused on the extensive exploitation of local sheep breeds, such as Churra, Latxa (which consists of two varieties, Cara Negra and Cara Rubia) and Manchega, which are highly adapted to the different local conditions, maximizing the use of local resources. In the last 20 years, however, some foreign specialized dairy sheep breeds, such as Assaf, Awassi, and Lacaune, are, to some extent, replacing the local breeds in semi-intensive and intensive farms [3] because of their higher milk yield potential. In particular, Assaf has the most significant impact on the Spanish and Portuguese dairy sheep industry [4]. 

For all the above-mentioned breeds, the genetic improvement for milk production is performed by the corresponding breeder association through official breeding programs, in which artificial insemination (AI) becomes a fundamental tool of progeny testing. AI plays a significant role because it is directly related to fertility and is the primary reproductive tool used for genetic improvement by introducing genes from superior sires into flocks [5]. Hence, AI is one of the most powerful biotechnological reproductive methods used in most breeding programs of livestock species.

In relation to AI, it is important to study the characteristics of semen production and semen quality. The number of doses produced per ram ejaculate depends on the volume, sperm concentration, and motility of the sperm, which are mainly affected by environmental and genetic factors [6]. 

To increase the efficiency of the dairy sheep AI centers, studies focused on the assessment of the environmental factors affecting semen production and the estimation of genetic parameters for seminal production and quality traits are required. In cattle, different authors have reported moderate heritabilities for some semen traits [5,7], suggesting the potential for genetic improvement not only for male reproduction traits but also for female reproductive traits, which are generally difficult to improve through direct selection [8]. 

In sheep, the limited studies addressing the estimation of genetic parameters for ram semen traits in different breeds show a wide variation [6,9,10], highlighting the importance of studying these traits for individual breeds. The estimation of genetic parameters is relevant not only to assess the possibility of the genetic improvement of semen traits but also to understand the interactions among the different traits and, therefore, help define the most efficient selection strategy and avoid undesired consequences of selection [5]. Finally, the inclusion of seminal traits in the male selection indices could be an aspect to consider and investigate in sheep breeds.

Given these considerations, the present study aimed to estimate genetic parameters for traits related to semen production and quality in Spanish dairy sheep breeds. For this purpose, four local breeds (Churra, Manchega, Latxa Cara Negra, and Latxa Cara Rubia) and one foreign highly productive breed (Assaf) were analyzed. Although the analyses here presented have been performed individually for each breed, this study provides an opportunity to assess the genetic parameters for semen traits across different breeds.

## 2. Materials and Methods 

### 2.1. Animals and Data

Three AI centers provided records for semen production and quality traits measured in five Spanish dairy sheep breeds: Ovigen (Centers for the Selection and Animal Breeding of Sheep and Goats of Castilla y León) for the Churra (CHU) and Assaf (ASS) breeds; Ardiekin, S.L. for the Latxa Cara Negra (LCN) and Latxa Cara Rubia (LCR) breeds; and Cersyra (Regional Centers of Reproduction and Animal Breeding of Castilla-La Mancha) for the Manchega (MAN) breed. According to the breeding program of the different breeds under study, a certain number of young males (the offspring of the best ewes from genetically connected flocks and of the best rams from the AI center) are handled in the AI center every year. After a training process for semen collection, young males that do not show mounting problems and are free from certain diseases are used as semen donors.

In each AI center, measurements of the traits of interest were obtained during the routine semen collection by artificial vagina and preparation of doses for AI. All the ejaculates were obtained by the same team of collectors at each AI center and a pool of 1 (all studied breeds mounted at least once) to 2 successive ejaculates obtained over a 2–5 min period (in all breeds except MAN, in which the rams usually mounted only once a day) was evaluated immediately after collection. For each pool, the total volume (VOL) was measured using a graduated collection tube (mL). After that, a sample of each pool was used to measure two other traits: semen concentration (SC), using a standard spectrophotometer (10^6^ spermatozoa per mL), and mass motility (MOT), which was assessed subjectively by examining the undiluted unstained semen sample under a microscope according to a wave motion continuous scale, from 0 (no motion) to 5 (numerous rapid and vigorous waves). The semen samples analyzed in this study were collected from the five dairy sheep breeds between 2003 and 2019 and belonged to a total of 3289 rams distributed among the five considered breeds as follows: 424 from the ASS rams, 497 CHU rams, 685 LCN rams, 655 LCR rams, and 1228 MAN rams.

From the initial raw dataset of semen measurements, records from animals whose age at semen collection was less than 10 months and greater than 150 months were excluded from the study. Additionally, all the measurements belonging to ejaculate with a motility score below 3 were removed from the database. This value (mass motility below 3) is the minimum quality threshold that AI centers usually use to consider an ejaculate suitable for insemination. After data filtering, the number of ejaculates available for each of the studied breeds, and used for subsequent analyses, were 19,938, 13,789, 16,342, 15,086, and 31,637 for ASS, CHU, LCN, LCR and MAN respectively. These records corresponded to the following number of rams per breed: 424 ASS, 497 CHU, 685 LCN, 653 LCR, and 1228 MAN for the first mount (Table 1). Likewise, the number of ejaculates for the second mount was 5223, 5254, 1838, and 875, corresponding to 367 ASS, 445 CHU, 452 LCN, and 317 LCR rams (see descriptive statistics for the second mount in Table S1).

Finally, the pedigree information was collected from different breed associations. All the known generations were used, generating a total coancestry matrix of 1969, 3902, 3866, 3089, and 3202 individuals in the ASS, CHU, LCN, LCR, and MAN breeds, respectively.

### 2.2. Statistical and Genetic Parameter Estimation

The three dependent variables studied (VOL, SC, and MOT) were analyzed individually for each breed. First, an initial general linear model (GLM) analysis was performed with R software version 3.6.1 [11] to evaluate the influence of the following fixed factors: the birth flock, the combined effect of the season and year of collection, the age of the male at collection and the number of consecutive mounts. Considering that most of the studied effects were highly statistically significant (*p* < 0.001) for all the traits and breeds (with only the age of the male at collection not significant for the VOL trait in ASS and CHU breeds) (Table S2), all of them were included in the subsequent estimation of the genetic parameters.

The estimates of the (co)variance components, heritabilities, and genetic correlations were obtained with multiple-trait animal models using the average information REML (restricted maximum likelihood) method by the BLUPF90 family of programs [12]. The data preparation was carried out with the renumf90 program from the same authors. The multiple-trait model for the studied dataset was as follows:

*Y_ijklmn_* = *F_i_* + *SY_j_* + *AC_k_* + *J_l_* + *RPE_m_* + *a_n_* + *r_ijklmn_*, (1)

where *Y_ijklmn_* are the individual trait values for VOL, SC, and MOT; F_i_ is the fixed effect of the flock of birth (ASS = 51 levels, CHU = 62, LCN = 81, LCR = 60, and MAN = 96); *SY_j_* is the fixed combination effect of the season (following the classification of Anel et al. [13] and year of collection (ASS = 41, CHU = 39, LCN = 57, LCR = 57, and MAN = 15 levels); *AC_k_* is the fixed effect of the age of the male at the time of the sperm collection (5 levels for all the breeds) and was classified as follows: class 1 for rams from 10 to 24 months, class 2 for rams from 25 to 36 months, class 3 for rams from 37 to 48, class 4 for rams from 49 to 60, and class 5 for rams from 61 months onwards; *J_l_* is the fixed effect of the number of consecutive mounts with 2 levels in the ASS, CHU, LCN, and LCR breeds. For the MAN breed, as the rams usually mounted only once a day, the *J_l_* effect included in the model referred to the mounting regime or interval between consecutive mounts expressed in days (from 1 to 8); *RPE_m_* is the random permanent environmental effect (ASS = 1 to 424, CHU = 1 to 497, LCN = 1 to 685, LCR = 1 to 655 and MAN = 1 to 1228); *a_n_* is the random additive genetic effects of the animal (ASS = 1969, CHU = 3902, LCN= 3866, LCR = 3089 and MAN = 3202); and *r_ijklmn_* is the random residual effect.

The variability in the measurements made on the same subject can be ascribed only to errors due to the measurement process itself. Hence, considering the different components of the phenotypic variance previously estimated through the multiple-trait model implemented for each breed, repeatability estimates were calculated for the different traits as follows:

(2) $$\frac{\sigma_{a\;}^{2}+\;\sigma_{RPE\;}^{2}}{\sigma_{p\;}^{2}},$$

where *σ_a_^2^* and *σ_RPE_^2^* are the variance estimates for the additive genetic and random permanent environmental effects, respectively, and ***σ_p_^2^*** is the phenotypic variance described as the sum of the additive genetic, random permanent environmental and residual variance effects.

## 3. Results

The descriptive statistics of the semen traits for the five breeds are shown in Table 1. The ASS breed had the highest mean value for two of the three traits studied, with values of 1.36 mL and 4.87 for the VOL and MOT traits, respectively. The ASS breed had the most extensive standard deviations for the VOL (0.54) and SC (1320.50) traits among the five breeds. The MAN breed showed the highest mean value for the SC (4513.80) trait and the largest standard deviation for the MOT trait (0.44). The LCR and LCN breeds presented very similar results for each of the studied traits. The phenotypic distributions observed for each trait and breed are provided in Supplementary Material (Figures S1–S3). As can be observed, the distributions for VOL and SC were very similar across breeds and close to normality, whereas the MOT distributions were not normal.

The estimates of the genetic, permanent environmental, and residual variances and repeatability are presented in Table 2. In general, the additive genetic variances estimated for the seminal traits were of similar magnitude among breeds, except for those estimated for the LCR breed, for all the traits, and the LCN breed, for SC and MOT traits, which were remarkably lower. The ASS breed showed the highest additive genetic variance for the VOL (0.030) and SC (281,870) traits, whereas the highest additive genetic variance for the MOT trait was found in CHU (0.017). The LCN and LCR breeds obtained the lowest additive genetic variances for all the traits. The repeatability estimates were similar among the breeds for VOL and SC, ranging between 0.211–0.291 for VOL and 0.270–0.303 for SC, whereas for MOT, the estimates obtained for the LCN and LCR (0.096 and 0.077, respectively) and MAN (0.079) breeds were notably lower than those estimated for ASS and CHU (0.197 and 0.151, respectively).

Regarding the genetic parameters presented in Table 3 and considering the three traits under study, we observed that the ASS, LCR, and MAN breeds showed similar heritability estimates (Table 3), with SC being the most heritable trait (0.20, 0.11, and 0.15, respectively). The VOL trait showed intermediate heritability estimates (0.12, 0.08, and 0.11, respectively), and the MOT trait showed estimates close to zero (0.03, 0.01, and 0.03, respectively). In the CHU breed, the SC trait showed similar heritability estimate (0.18) to those found in ASS and MAN, whereas the estimates obtained for VOL and MOT were substantially higher than those found in the other breeds (0.20 and 0.11, respectively). The heritability estimate for the VOL trait in CHU was the highest heritability reported in this work. Finally, the LCN breed showed similar trends of heritability estimates to those of CHU, although with lower values, with the VOL trait showing the highest heritability value (0.16) within this breed.

Among the genetic correlations estimated in this work (Table 3), only those estimated for the MAN breed showed acceptable accuracy, as very large standard errors (SE) were observed for the rest of breeds estimates. Focusing on MAN, moderate and negative correlations between the VOL trait and the two other traits were observed (−0.49 and −0.29 with SC and MOT, respectively), whereas a moderate positive correlation was estimated between the SC and MOT traits (0.32).

In the ASS and CHU breeds, moderate and positive phenotypic correlations were found for the SC-VOL and SC-MOT trait pairs (with a range from 0.16 to 0.23), with close to zero negative estimates between MOT and VOL; in the LCN and LCR breeds, the MOT-SC phenotypic correlation was positive (0.16 and 0.18, respectively), whereas all the phenotypic correlations in MAN were low (Table 3).

## 4. Discussion

Dairy sheep are traditionally reared in Mediterranean areas on a wide range of production systems. In the last 30 years, dairy sheep systems have kept their dual purpose and have maintained an income characterized by a 65–75% contribution from milk and 25–35% from meat. The efficiency of selection for milk traits is now whetting interest in new breeding goals, which are relevant to satisfying consumer demand with other products, such as quality-labeled cheeses, and “healthy” food products [3].

Milk yield is one of the most important economic traits in dairy sheep, but in practice (for genetic improvement), fertility is also a critical trait for the economic efficiency of dairy sheep flocks [14]. According to different studies in other species, semen quantity and quality are the main factors affecting the fertility of rams used for AI [15,16]. AI is becoming increasingly essential within sheep breeding programs, allowing the dissemination of the genetic gain to the whole population. Reproductive efficiency has become a key factor to consider because improved reproductive success could result in greater genetic progress for selected traits [17]. Therefore, the selection of rams should be focused not only on milk production traits but also on semen traits in which the estimation of genetic parameters is a required step.

The dataset compiled through this study has provided the opportunity to analyze different seminal traits rarely studied in the ovine species and to compare phenotypic and genetic parameters among five of the most important Spanish dairy sheep breeds. Traits such as volume, sperm concentration, and sperm motility are relevant traits to analyze in the ejaculate of rams.

In general, the mean values reported here for these traits were very similar to those obtained in other sheep breeds [6,18]. The mean value observed for the MOT trait in the MAN breed (3.61) was low compared with that of the rest of breeds analyzed here and also stood out compared with French breeds [6]. We think that the deviation from the normality and the subjectivity of the MOT has affected the accuracy of the genetic parameters estimated here for this trait, as will be discussed below.

Seminal traits are affected by environmental, management, and genetic effects and physiological status [6]. In our case, three AI centers located in different regions of Spain provided semen production data, and the main environmental factors affecting sperm production were collected at each center. In this study, because the analyses were performed individually for each breed and the different centers analyzed different breeds, the reported differences might be due not only to the breed factor but also to the different criteria to consider an ejaculate suitable for AI at each AI center. For example, for the MAN breed, a sequential evaluation of ejaculates is carried out in such a way that a minimum volume is required for an ejaculate; those ejaculates that pass this criterion are evaluated for motility, and once they pass this criterion, the concentration is measured. Therefore, all the ejaculates with concentration data will have volume and motility data above the respective thresholds. On the other hand, the AI center that analyzed the samples from LCN and LCR breeds implements more restrictive criteria to declare an ejaculate suitable for AI (a mass motility value of 4 and 0.3 for volume are required as minimum values). Hence, these breeds show a relatively low variability compared with the rest of breeds, especially for the SC and MOT traits. In the case of the breeds analyzed in the same AI centers, CHU-ASS and LCN-LCR, the differences reported between them are more likely to reflect genuine differences due to the breed effect. In any case, we acknowledge that the use of these quality thresholds can introduce certain biases on the genetic parameter estimates reported here (e.g., a lower heritability due to the artificially reduced phenotypic variance). Hence, the ideal situation for this kind of study would be that all semen quality measures were recorded without any censorship in all the AI centers; but this state is unrealistic in practical conditions. The impact of the thresholds will depend on the proportion of samples that are below the corresponding threshold in each breed and also the change in the variable distributions when including or discarding some of the data.

The effects of flock and the interaction of collection season–year were significant for all the traits and breeds. Our results are in agreement with those obtained by Anel et al. [13] in the Churra breed, where the farm effect was found to have a major influence on ewes’ fertility. The season and year of collection were also found to be significant environmental factors, probably related to the seasonal reproductive behavior of the ovine species. As expected, all breeds analyzed in this work showed the highest values for the three studied traits at the favorable reproductive season (i.e., the months from September to February). This agrees with observations reported in other sheep breeds [19]. These two factors, whose relevance to semen traits has been highlighted by other authors, involve many environmental factors, such as animal care, climatic changes, temperature, feed quality, and length of day [20,21]. Another critical effect influencing semen production and quality detected here was the age of the male at the collection time. This factor was significant for all the traits and breeds, except for VOL in ASS and CHU. In this case, the age factor showed a variable effect on the studied traits depending on the breed. For example, the highest values for the three studied traits were observed, for the ASS, CHU, and MAN breeds, in rams with age between 37 and 60 months; and for the LCN and LCR breeds, for rams included in two different age groups (10–24 months and 49–60 months). The common effect of age across all the breeds was an age-related decrease in semen trait values (rams with more than 60 months), which is in agreement with observations reported in Lacaune and Manech Tête Rousse breeds [6]. Additionally, in Austrian Simmental bulls, a decrease in semen quality traits, without effect on the VOL trait, has also been reported to be associated with increased age [22]. Finally, both the mounting effect (in ASS, CHU, LCN, and LCR breeds) and mounting regime effect (only in MAN breed) were significant for all the traits. In our analysis, a maximum of two ejaculates per animal was collected every 2–5 min (except in the MAN breed, in which only one ejaculate per animal was routinely collected). Comparing the results obtained here in the different mountings, the mean values for VOL and SC traits in mount 2 were, in general, lower than in mount 1, whereas slightly higher MOT mean values were observed in mount 2 compared to mount 1. David et al. [6] also found a significant influence of the mounting effect in the Lacaune and Manech Tête Rousse breeds, reporting higher motility and a lower number of spermatozoa with a shorter interval between two collections. They also suggested that there could be an optimum interval, which ensures a higher quantity of highly motile spermatozoa, but this question needs to be further investigated. For the MAN breed, with only one mount per collection, the mounting regime effect, measured as the interval in days between two consecutive semen collections, was important. Thus, an interval of 3 days was found to be optimum for the three semen traits assessed in this study. Lower intervals (1–2 days) resulted in a decrease in semen production and quality, whereas intervals above 3 days did not increase the trait values.

The highest additive genetic variances were found for the VOL and SC traits in the ASS breed and the MOT trait in the CHU breed. The large additive genetic variances observed in ASS can be explained by the fact that this breed has been recently created by the absorption of several Spanish breeds, the large current census of the Assaf population and its recent breeding program (initiated in 2005), compared with that of the other studied breeds.

The coefficients of repeatability describing the degree of similarity between consecutive measurements affected by random environmental factors were also estimated. The SC trait showed the highest repeatability value in all the breeds studied. The repeatability values found in this work were lower than those estimated in other sheep breeds [6] and bovine breeds [20,21]. The low to moderate repeatability values obtained for the analyzed semen traits suggest that first collection records are not good indicators of the future reproductive yield of AI rams.

The heritability estimates obtained in this study are similar to those described in the literature. For example, David et al. [6] reported similar results in two French sheep breeds ranging from 0.07 (for the MOT trait) to 0.27 (for the VOL and SC traits) in the Lacaune breed and from 0.02 (for the MOT trait) to 0.33 (for the VOL trait) in the Manech Tête Rousse breed. We think the deviation from the normality observed for the MOT trait, and the subjectivity linked to the measurements of this trait, can explain why this is the trait for which the lowest heritability estimates were observed (four out of the five breeds had heritability estimates lower than 0.04, only CHU reached an estimate higher than 0.1). Our data are also comparable to those published for other species. For example, Li et al. [16] reported similar heritabilities in pigs (Landrace boars), ranging from 0.11 to 0.23 (for the MOT and SC traits, respectively), and Karoui et al. [7], showed a similar pattern, obtaining heritabilities from 0.16 to 0.22 (for the MOT and VOL traits, respectively) in Holstein bulls. In contrast, other researchers found higher heritabilities for the MOT trait, surpassing those found in the VOL or SC traits. For example, in Ethiopian sheep, Rege et al. [9] found values of 0.32 and 0.27 for the MOT trait in 9 and 12-month-old rams, respectively. For Holstein bulls, Druet et al. [5] obtained a heritability of 0.43, and Li et al. [16], found values of 0.26 and 0.42 in Yorkshire and Duroc pig breeds, respectively. Heritability estimates found in the present study and the variability found in different breeds and species point towards the opportunity for genetic improvement of some semen traits through selective breeding. In any case, from a practical point of view, before considering the improvement of these traits through selection, it would be necessary to estimate the genetic correlations between the semen traits and the current selection objectives of the breeding programs of these breeds, which are mainly focused on dairy-related traits.

Understanding the relationship between the different seminal traits is very informative for the efficient development of selection strategies aimed at the genetic improvement of the considered target traits. Generally, similar values of phenotypic correlations to those estimated here for VOL, SC, and MOT were found in the literature [6]. The low phenotypic correlations obtained for some of the breeds in this and other studies [9] may indicate that some environmental factors, particularly when considering a wide range of time, influencing the studied traits are not controllable by the AI centers (e.g., seasonality temperature, dryness). It should also be considered that for some of these environmental effects, for example, related to the mounting regimen, ovine AI centers may implement a more variable routine than other species AI centers (e.g., cattle, pig).

This influence of non-controllable environmental factors might explain the high standard error of the genetic correlations reported for most of the breeds in this study. Only for the MAN breed did genetic correlation estimates among the semen traits show adequate accuracy (low SE). This may be due to the higher number of animals analyzed in the MAN breed (1200 vs. 500 in the other breeds) and to a more suitable family structure for the determination of genetic relationships. These are key factors influencing the precision of the genetic correlation estimates [23]. Hence, focusing on the MAN estimates, the moderate negative genetic correlation (−0.49) observed between VOL and SC agrees with the average estimates reported, for both young and adult animals, for this pair of traits in Lacaune and Manech Tête Rousse breeds [6] and Ethiopian highland sheep [9]. This agrees with the expected relationship between traits of this nature (volume/concentration), which would determine the need for considering both traits when trying to improve semen production and quality in rams. For the other two trait pairs involving the MOT trait, which showed a non-normal distribution, the genetic correlations showed remarkably high SE for MAN, and also for the other breeds. When comparing the published genetic correlations for SC-MOT and VOL-MOT, we found important discrepancies among studies, even different for breeds and age groups analyzed in the same study [6,9]. Whereas for the VOL-MOT pair, the genetic correlation estimate reported here for MAN (−0.29) shows the same direction but higher magnitude than those reported in French breeds (range: −0.03 to −0.09) [6], this estimate clearly differs from that reported in Ethiopian sheep (0.51) [9]. The moderate positive genetic correlation that we found for the SC-MOT pair (0.32) agrees with the estimates reported for young animals of the Manech Tête Rousse (0.44) [6] and Ethiopian highland breeds (0.58) [9], but not in the Lacune breed (0.04–0.07) [6]. All these discrepancies are likely to be explained by the high level of subjectivity when measuring the MOT trait in AI centers (as shown in Figures S1–S3), which is also directly related to the low heritability estimates reported here for this trait, not only in MAN but in all the studied breeds. Because of the relevance of the MOT trait in sheep fertility [24], we consider that if the moderate positive correlation reported here and by Davis et al. [6] for the SC-MOT pair (0.32 and 0.44, respectively) is confirmed in MAN by future studies, the MOT trait could be improved by indirect selection using the SC traits as a proxy.

## 5. Conclusions

In the present study, genetic parameters for three semen traits were estimated in Spanish dairy sheep breeds. The heritability estimates reported were, in general, low to moderate, suggesting that improvement of these traits may be achieved by genetic selection. The genetic correlations among the three semen traits showed adequate precision in the MAN breed. Considering the difficulties of fully understanding the genetic relationships between semen quality traits based on existing datasets (prospective studies), future efforts should be focused on experiments specifically designed to maximize control of the environmental factors influencing these traits.

## Figures and Tables

**Table 1:** Descriptive statistics for the semen traits analyzed in the present work for five Spanish dairy sheep breeds for the first mounting.

Trait Breed	N° Rams	N° Records	Mean	Standard Deviation	Minimum	Maximum
VOL						
ASS	424	19,938	1.36	0.54	0.1	4.7
CHU	497	13,787	1.02	0.39	0.1	3.7
LCN	685	16,342	0.99	0.35	0.3	3.4
LCR	653	15,086	0.97	0.34	0.3	3.0
MAN	1228	31,637	1.02	0.38	0.1	3.5
SC						
ASS	424	19,892	4163.44	1320.50	121	9701
CHU	497	13,754	4031.71	1245.15	201	9999
LCN	685	16,342	3844.51	667.54	2355	6304
LCR	653	15,086	3625.02	642.06	2353	6019
MAN	1228	31,637	4513.80	923.20	970	8540
MOT						
ASS	424	19,938	4.87	0.39	3	5
CHU	497	13,789	4.82	0.43	3	5
LCN	685	16,342	4.86	0.06	4	5
LCR	653	15,086	4.85	0.06	4	5
MAN	1228	31,637	3.61	0.44	3	5

Note. ASS: Assaf; CHU: Churra; LCN: Latxa Cara Negra; LCR: Latxa Cara Rubia: MAN: Manchega. VOL: volume of ejaculate (mL); SC: sperm concentration (×10^6^ spermatozoa/mL); and MOT: mass motility (0–5).

**Table 2:** Estimates of the genetic parameters for the three semen traits for the five Spanish dairy sheep breeds considered. The three traits studied were the volume of ejaculate (VOL, mL), the sperm concentration (SC: ×10^6^ spermatozoa/mL), and the mass motility (MOT; linear scale: 0–5).

Breed	Trait	σ_a_^2^	σ_RPE_^2^	σ_r_^2^	σ_p_^2^	R
ASS	VOL	0.030	0.022	0.194	0.246	0.211 (0.02)
	SC	281,870	114,540	1,074,500	1,470,900	0.270 (0.02)
	MOT	0.004	0.022	0.106	0.132	0.197 (0.01)
CHU	VOL	0.026	0.013	0.095	0.134	0.291 (0.02)
	SC	200,450	142,130	787,620	1,130,200	0.303 (0.02)
	MOT	0.017	0.007	0.136	0.159	0.151 (0.01)
LCN	VOL	0.020	0.011	0.091	0.122	0.251 (0.02)
	SC	32,420	61,417	223,450	317,290	0.296 (0.02)
	MOT	9.5 × 10^−5^	0.000	0.003	0.003	0.096 (0.01)
LCR	VOL	0.009	0.020	0.087	0.116	0.252 (0.02)
	SC	33,144	58,266	209,160	300,570	0.304 (0.02)
	MOT	4.1 × 10^−5^	0.000	0.003	0.003	0.077 (0.01)
MAN	VOL	0.016	0.022	0.104	0.142	0.268 (0.01)
	SC	119,150	106,400	559,300	784,850	0.287 (0.01)
	MOT	0.004	0.007	0.128	0.139	0.079 (0.01)

Note. ASS: Assaf; CHU: Churra; LCN: Latxa Cara Negra; and LCR: Latxa Cara Rubia. Additive genetic variance (σa^2^), permanent environmental variance (σRPE^2^), residual variance (σr^2^), phenotypic variance (σp^2^), and repeatability (R, with standard error) estimate.

**Table 3:** Genetic parameters for three semen traits analysed in five Spanish ovine dairy breeds. For each population (ASS, CHU, LCN, LCR, and MAN) heritabilities are presented in bold on the diagonal of the corresponding breed row. Also for each breed, the genetic and phenotypic correlations among the analysed traits are indicated above and below, respectively, the corresponding diagonal. The SE of each estimate is shown in brackets.

Breed	Trait	VOL	SC	MOT
ASS	VOL	**0.12 (0.04)**	−0.25 (0.25)	−0.33 (0.92)
	SC	0.22 (0.01)	**0.19 (0.05)**	0.20 (0.65)
	MOT	−0.03 (0.01)	0.20 (0.01)	**0.03 (0.03)**
CHU	VOL	**0.20 (0.04)**	0.02 (0.20)	-0.12 (0.20)
	SC	0.23 (0.01)	**0.18 (0.05)**	0.34 (0.21)
	MOT	−0.04 (0.01)	0.16 (0.01)	**0.11 (0.03)**
LCN	VOL	**0.16 (0.04)**	−0.20 (0.36)	0.37 (0.50)
	SC	0.06 (0.01)	**0.10 (0.04)**	−0.04 (0.87)
	MOT	0.01 (0.01)	0.18 (0.01)	**0.03 (0.02)**
LCR	VOL	**0.08 (0.03)**	−0.71 (0.44)	−0.37 (2.05)
	SC	0.02 (0.01)	**0.11 (0.04)**	0.06 (0.81)
	MOT	0.02 (0.01)	0.16 (0.01)	**0.01 (0.01)**
MAN	VOL	**0.11 (0.05)**	−0.49 (0.13)	−0.29 (0.18)
	SC	0.06 (0.01)	**0.15 (0.06)**	0.32 (0.16)
	MOT	0.01 (0.01)	0.04 (0.01)	**0.03 (0.03)**

Note. ASS: Assaf; CHU: Churra; LCN: Latxa Cara Negra; and LCR: Latxa Cara Rubia. VOL: volume of ejaculate (ml); SC: sperm concentration (x10^6^ spermatozoa/mL); and MOT: mass motility (0–5).

## Supplementary Materials

The following are available online at https://www.mdpi.com/2076-2615/9/12/1147/s1, Table S1: Descriptive statistics for the semen traits analyzed in the present work for three Spanish dairy sheep breeds for the second mounting, Table S2: Significance of the fixed effects on the evaluated traits, Figure S1: The phenotypic distributions observed for the MAN breed in the three studied traits (VOL, SC, and MOT traits, respectively). Data provided by Cersyra center (Regional Centers of Reproduction and Animal Breeding of Castilla-La Mancha), Figure S2: The phenotypic distributions observed for the ASS and CHU breeds in the three studied traits (VOL, SC, and MOT traits, respectively). Data provided by Ovigencenter (Centers for the Selection and Animal Breeding of Sheep and Goats of Castilla y León), Figure S3: The phenotypic distributions observed for the LCN and LCR breeds in the three studied traits (VOL, SC, and MOT traits, respectively). Data provided by Ardiekin, S.L. center.

## References

[1] FAOSTAT F. (2018). Statistical Data.

[2] Toro-Mujica P., García A., Gómez-Castro A.G., Acero R., Perea J., Rodríguez-Estévez V., Aguilar C., Vera R. (2011). Technical efficiency and viability of organic dairy sheep farming systems in a traditional area for sheep production in Spain. Small Rumin. Res..

[3] Sanna S.R., Casu S., Carta A. Breeding programmes in dairy sheep. Proceedings of the 7th World Congress on Genetics Applied to Livestock Production.

[4] De La Fuente L.F., Gabiña D., Carolino N., Ugarte E. The Awassi and Assaf breeds in Spain and Portugal. Proceedings of the 57st Annual Meeting of the EAAP.

[5] Druet T., Fritz S., Sellem E., Basso B., Gérard O., Salas-Cortes L., Humblot P., Druart X., Eggen A. (2009). Estimation of genetic parameters and genome scan for 15 semen characteristics traits of Holstein bulls. J. Anim. Breed. Genet..

[6] David I., Druart X., Lagriffoul G., Manfredi E., Robert-Granié C., Bodin L. (2007). Genetic and environmental effects on semen traits in Lacaune and Manech tête rousse AI rams. Genet. Sel. Evol..

[7] Karoui S., Díaz C., Serrano M., Cue R., Celorrio I., Carabaño M.J. (2011). Time trends, environmental factors and genetic basis of semen traits collected in Holstein bulls under commercial conditions. Anim. Reprod. Sci..

[8] Berglund B. (2008). Genetic Improvement of Dairy Cow Reproductive Performance. Reprod. Domest. Anim..

[9] Rege J.E.O., Toe F., Mukasa-Mugerwa E., Tembely S., Anindo D., Baker R.L., Lahlou-Kassi A. (2000). Reproductive characteristics of Ethiopian highland sheep II. Genetic parameters of semen characteristics and their relationships with testicular measurements in ram lambs. Small Rumin. Res..

[10] Lagriffoul G., Robert-Granié C., Manfredi E., Bodin L., David I. (2009). Character process model for semen volume in AI rams: evaluation of correlation structures for long and short-term environmental effects. Genet. Sel. Evol..

[11] Team R.C. A Language and Environment for Statistical Computing. R Foundation for Statistical Computing, Vienna, Austria. https://www.R-project.org/.

[12] Misztal I., Tsuruta S., Lourenco D., Aguilar I., Legarra A., Vitezica Z. (2018). Manual for BLUPF90 Family of Programs.

[13] Anel L., Kaabi M., Abroug B., Alvarez M., Anel E., Boixo J.C., De La Fuente L.F., De Paz P. (2005). Factors influencing the success of vaginal and laparoscopic artificial insemination in churra ewes: A field assay. Theriogenology.

[14] Legarra A., Ramón M., Ugarte E., Pérez-Guzmán M.D. (2007). Economic weights of fertility, prolificacy, milk yield and longevity in dairy sheep. Animal.

[15] Zak L.J., Gaustad A.H., Bolarin A., Broekhuijse M.L.W.J., Walling G.A., Knol E.F. (2017). Genetic control of complex traits, with a focus on reproduction in pigs. Mol. Reprod. Dev..

[16] Li X., Jiang B., Wang X., Liu X., Zhang Q., Chen Y. (2019). Estimation of genetic parameters and season effects for semen traits in three pig breeds of South China. J. Anim. Breed. Genet..

[17] Jiménez-Rabadán P., Pérez-Guzmán M.D., Garde J.J., García-Alvarez O., Maroto-Morales A., Soler A.J., Fernández-Santos M., Ramón M. Genetic parameters for sperm morphometric traits in rams of Manchego sheep. Proceedings of the XVI Jornadas sobre Producción Animal.

[18] Carvajal-Serna M., Cortés-López H.A., Manrique-Perdomo C., Grajales-Lombana H.A. (2018). Evaluación de los parámetros de calidad seminal y cinemática espermática en tres razas ovinas de lana en condiciones de trópico alto colombiano. Rev. Med. Vet. (Bogota)..

[19] Belkadi S., Safsaf B., Heleili N., Tlidjane M., Belkacem L., Oucheriah Y. (2017). Seasonal influence on sperm parameters, scrotal measurements, and serum testosterone in Ouled Djellal breed rams in Algeria. Veterinary World.

[20] Mathevon M., Buhr M.M., Dekkers J.C.M. (2010). Environmental, Management, and Genetic Factors Affecting Semen Production in Holstein Bulls. J. Dairy Sci..

[21] Paldusová M., Kopec T., Filipcík R., Hosdek M., Máchal L. (2016). Effect of selected factors on qualitative and quantitative semen parameters of Czech Fleckvieh bulls. Acta Univ. Agric. Silvic. Mendelianae Brun..

[22] Fuerst-Waltl B., Schwarzenbacher H., Perner C., Sölkner J. (2006). Effects of age and environmental factors on semen production and semen quality of Austrian Simmental bulls. Anim. Reprod. Sci..

[23] Falconer D.S., Mackay T.F.C. (1996). Introduction to Quantitative Genetics.

[24] David I., Kohnke P., Lagriffoul G., Praud O., Plouarboué F., Degond P., Druart X. (2015). Mass sperm motility is associated with fertility in sheep. Anim. Reprod. Sci..

