# Supplementary material for: Estimation of the Genetic Parameters for Semen Traits in Spanish Dairy Sheep
Source: Animals (Basel). 2019 Dec 13;9(12):1147. doi: 10.3390/ani9121147 (PMC6940766; doi:10.3390/ani9121147)
Supplement: Supplementary file 1 [file animals-09-01147-s001.pdf]

SUPPLEMENTARY MATERIAL

ESTIMATION OF THE GENETIC PARAMETERS FOR SEMEN TRAITS IN SPANISH DAIRY SHEEP.

By: Rocío Pelayo, Manuel Ramón, Itsasne Granado-Tajada, Eva Ugarte, Malena Serrano, Beatriz Gutiérrez-Gil, Juan-José. Arranz.

Table S1. Descriptive statistics for the semen traits analysed in the present work for four Spanish dairy sheep breeds for the second mounting.

**Trait/breed**	**Nº rams**	**Nº records**	**Mean**	**Sd**	**Minimum**	**Maximum**
VOL						
ASS	367	5,223	0.95	0.37	0.2	4.7
CHU	445	5,254	0.81	0.29	0.1	3
LCN	452	1,838	0.93	0.40	0.3	3
LCR	317	875	0.91	0.44	0.3	3.8
SC						
ASS	367	5,200	3,625.55	1,101.11	279	8,018
CHU	445	5,233	3,519.01	1,038.02	201	8,257
LCN	452	1,838	3,860.85	683.74	2,355	5,827
LCR	317	875	3,591.05	633.32	2,353	5,433
MOT						
ASS	367	5,223	4.91	0.32	3	5
CHU	445	5,254	4.88	0.34	3	5
LCN	452	1,838	4.87	0.05	4.7	5
LCR	317	875	4.86	0.05	4.7	4.9

Note. ASS: Assaf; CHU: Churra; LCN: Latxa Cara Negra; and LCR: Latxa Cara Rubia. VOL: volume of ejaculate (ml); SC: sperm concentration (x10^6 spermatozoa/ml); and MOT: mass motility (0-5)

Table S2. Significance of the fixed effects on the evaluated traits.

	ASS			CHU			LCN			LCR			MAN		
**Fixed Effect**	**VOL**	**SC**	**MOT**	**VOL**	**SC**	**MOT**	**VOL**	**SC**	**MOT**	**VOL**	**SC**	**MOT**	**VOL**	**SC**	**MOT**
Flocks	***	***	**	***	***	**	***	***	***	***	***	***	***	***	***
Interaction season-year of collection	***	***	***	***	***	***	***	***	***	***	***	***	***	***	***
Age of male at collection	NS	***	***	NS	***	***	***	***	***	***	***	***	***	***	**
Mounts	***	***	***	***	***	***	***	***	***	***	***	*	-	-	-
Mounting regime	-	-	-	-	-	-	-	-	-	-	-	-	***	**	***

Note. ASS: Assaf; CHU: Churra; LCN: Latxa Cara Negra; and LCR: Latxa Cara Rubia. VOL: volume of ejaculate (ml); SC: sperm concentration (x10^6 spermatozoa/ml); and MOT: mass motility (0-5).

***= (p< 0.001); ** = (p<0.01); * = (p<0.05) and NS = (p>0.05).

SUPPLEMENTARY MATERIAL

ESTIMATION OF THE GENETIC PARAMETERS FOR SEMEN TRAITS IN SPANISH DAIRY SHEEP.

By: Rocío Pelayo, Manuel Ramón, Itsasne Granado-Tajada, Eva Ugarte, Malena Serrano, Beatriz Gutiérrez-Gil, Juan-José. Arranz.

Figure S1. The phenotypic distributions observed for the MAN breed in the three studied traits (VOL, SC and MOT traits, respectively). Data provided by Cersyra centre (Regional Center of Reproduction and Animal Breeding of Castilla-La Mancha).

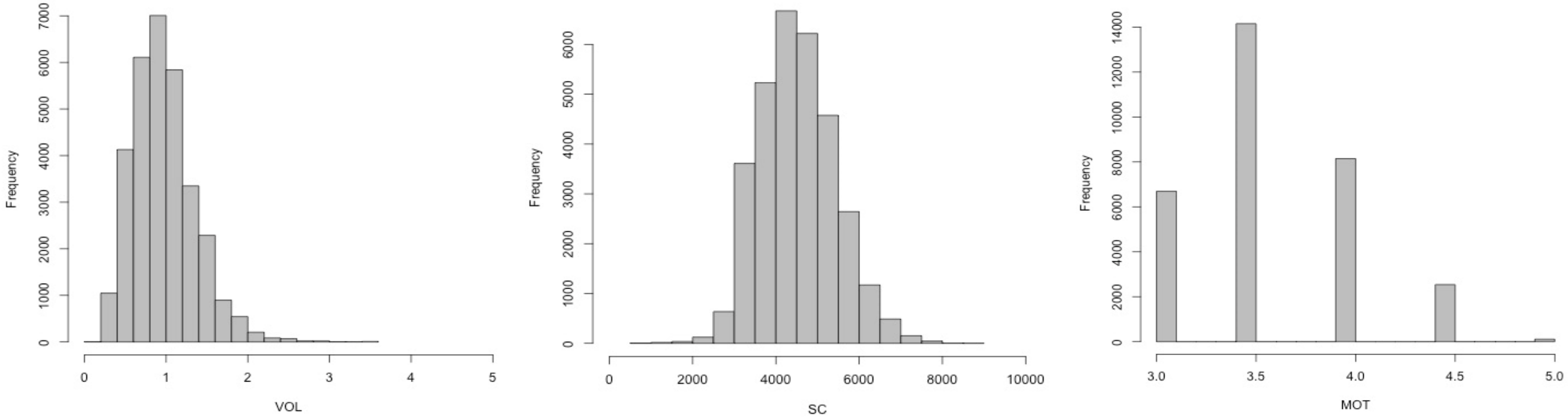

MAN breed

Figure S2. The phenotypic distributions observed for the ASS and CHU breeds in the three studied traits (VOL, SC and MOT traits, respectively). Data provided by Ovigen centre (Center for the Selection and Animal Breeding of Sheep and Goats of Castilla y León).

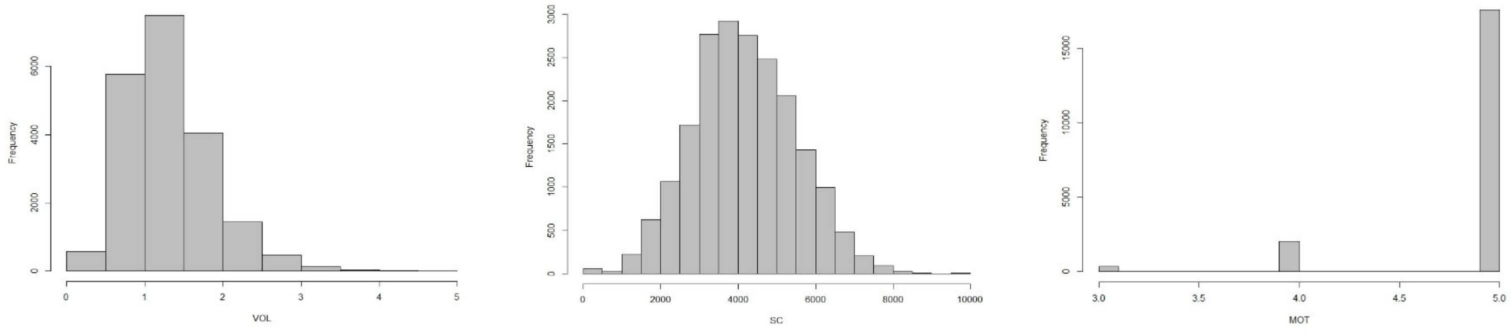

ASS breed

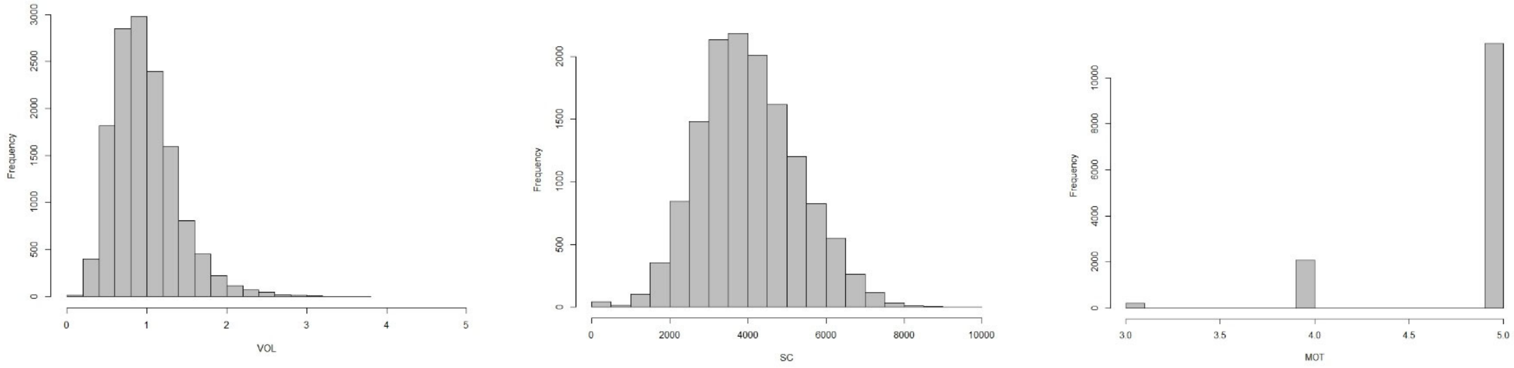

CHU breed

Figure S3. The phenotypic distributions observed for the LCN and LCR breeds in the three studied traits (VOL, SC and MOT traits, respectively). Data provided by Ardiekin, S.L. centre.

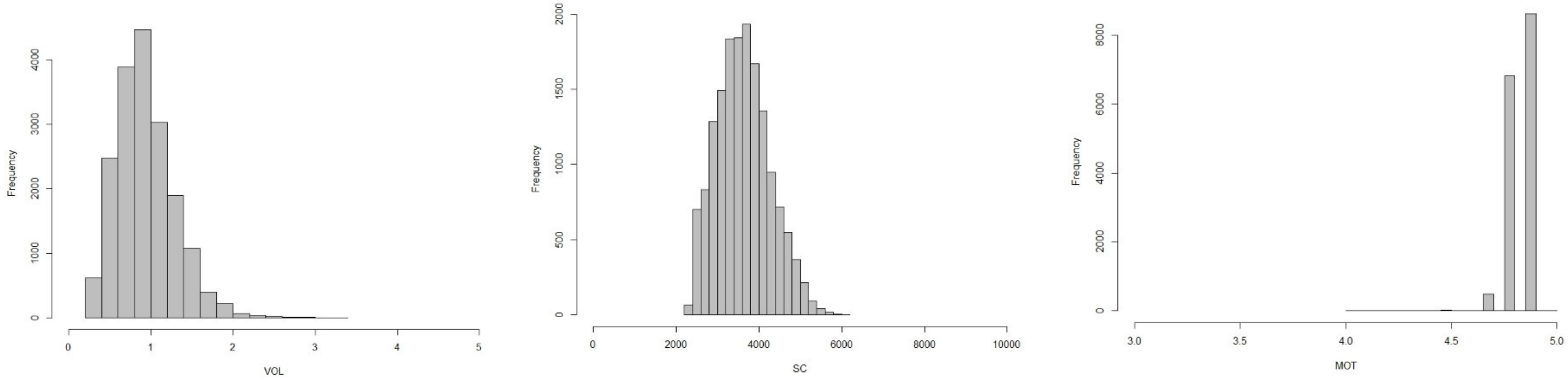

LCN breed

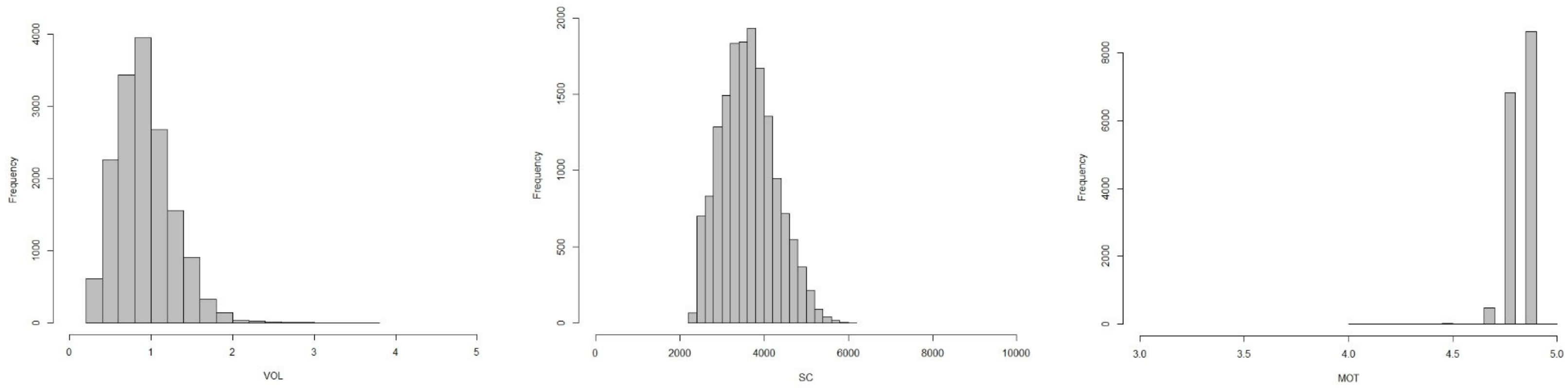

LCR breed
